# Users, Uses, and Effects of Social Media in Dietetic Practice: Scoping Review of the Quantitative and Qualitative Evidence

**DOI:** 10.2196/jmir.9230

**Published:** 2018-02-20

**Authors:** Audrée-Anne Dumas, Annie Lapointe, Sophie Desroches

**Affiliations:** ^1^ Institute of Nutrition and Functional Foods School of Nutrition Laval University Quebec City, QC Canada

**Keywords:** social media, diet, review

## Abstract

**Background:**

Social media platforms are increasingly used by registered dietitians (RDs) to improve knowledge translation and exchange in nutrition. However, a thorough understanding of social media in dietetic practice is lacking.

**Objective:**

The objective of this study was to map and summarize the evidence about the users, uses, and effects of social media in dietetic practice to identify gaps in the literature and inform future research by using a scoping review methodology.

**Methods:**

Stages for conducting the scoping review included the following: (1) identifying the research question; (2) identifying relevant studies through a comprehensive multidatabase and gray literature search strategy; (3) selecting eligible studies; (4) charting the data; and (5) collating, summarizing, and reporting results for dissemination. Finally, knowledge users (RDs working for dietetic professional associations and public health organizations) were involved in each review stage to generate practical findings.

**Results:**

Of the 47 included studies, 34 were intervention studies, 4 were descriptive studies, 2 were content analysis studies, and 7 were expert opinion papers in dietetic practice. Discussion forums were the most frequent social media platform evaluated (n=19), followed by blogs (n=13) and social networking sites (n=10). Most studies targeted overweight and obese or healthy users, with adult populations being most studied. Social media platforms were used to deliver content as part of larger multiple component interventions for weight management. Among intervention studies using a control group with no exposition to social media, we identified positive, neutral, and mixed effects of social media for outcomes related to users’ health behaviors and status (eg, dietary intakes and body weight), participation rates, and professional knowledge. Factors associated with the characteristics of the specific social media, such as ease of use, a design for quick access to desired information, and concurrent reminders of use, were perceived as the main facilitators to the use of social media in dietetic practice, followed to a lesser extent by interactions with an RD and social support from fellow users. Barriers to social media use were mostly related to complicated access to the site and time issues.

**Conclusions:**

Research on social media in dietetic practice is at its infancy, but it is growing fast. So far, this field of research has targeted few social media platforms, most of which were assessed in multiple-component interventions for weight management among overweight or obese adults. Trials isolating the effects and mechanisms of action of specific social media platforms are needed to draw conclusions regarding the effectiveness of those tools to support dietetic practice. Future studies should address barriers and facilitators related to the use of social media written by RDs and should explore how to make these tools useful for RDs to reach health consumers to improve health through diet.

## Introduction

With the increasing worldwide prevalence of obesity [1] and its related comorbidities [2,3], effective and low-cost approaches that can improve health behaviors, such as those related to diet, are needed to improve health and well-being in populations. The advent of Web 2.0 [4] has triggered a revolution in the way patients access health information for their health management [5,6] and provide opportunities for population-wide promotion of healthy behaviors. Social media is a broad example of Web 2.0 and refers to Internet-based platforms devoted to blogging, social networking, collaborative writing projects, content communities, and virtual social worlds [7].

Social media platforms are novel avenues with high reach potential of dissemination that can be used by health care professionals to improve knowledge translation of evidence-based health information to health consumers and patients. The growing use of social media by patients and health professionals has been widely advocated in the scientific literature [8-10]. A survey of 195 registered dietitians (RDs) and dietetic students conducted by the Dietitian Connection network in Australia found that almost all (97%) of RDs use social media, Facebook being the platform of predilection followed by Instagram, illustrating that visual imagery has significantly gained in popularity among RDs for showcasing food and recipes [11]. Furthermore, social media represents valuable additions to traditional face-to-face clinical encounters to deliver behavioral interventions [12] notably to support long-term and sustained dietary behavior change efforts for chronic disease management and prevention [13].

Social media can be used for numerous purposes in dietetic practice, including public health. Social media has been used to broaden the scope of nutrition education program by using different social media platforms (Facebook, Twitter, and Pinterest) to disseminate actionable messages [14,15]. Social media also provides a promising way to deliver dietary behavior change interventions [16-18].

Dietetic professional associations have recognized the role of social media RDs’ professional practice [19] and feature a repertoire of their members who are active on social media such as Twitter [20] and blogs [21,22]. However, much remains unknown in the scientific literature about social media in dietetic practice and whether they can help health consumers make informed decisions to improve health through diet.

To fill this gap in knowledge, we aimed to answer the following research question: What evidence is provided about the users, uses, and effects of social media in dietetic practice? The specific research questions were as follows:

1. Who is using social media in dietetic practice?
2. What are the purposes of social media in dietetic practice?
3. What are the effects of interventions using social media in dietetic practice on food- and nutrition-related outcomes?
4. What are the barriers and facilitators that could influence the use of social media in dietetic practice?
5. What are the research gaps in this literature to inform future research?

## Methods

### Knowledge Synthesis Methodology

Our research objectives were addressed using the scoping review methodology, which is a type of knowledge synthesis that aims to map rapidly the key concepts underpinning a research area and the main sources and types of evidence available [23]. We formulated our protocol [24] using the methodology proposed by Arksey and O’Malley [23] and taking into account recommendations by Levac et al [25]. All steps were iterative to ensure full understanding of the content and extent of the literature. A summary of our 6-stage methodology follows.

### Stage 1: Identifying the Research Question

Studies were included if they reported primary questions focused on the users, uses, or effects of social media on food- and nutrition-related outcomes. On the basis of Kaplan and Heanleins’s classification scheme [7], we defined *social media* as “a group of Internet-based applications that build on the ideological and technological foundations of Web 2.0, and that allow the creation and exchange of user-generated content,” including the following platforms: collaborative projects (eg, wikis), blogs and microblogs (eg, Twitter), content communities (eg, Pinterest), social networking sites (eg, Facebook), and virtual social worlds (eg, Second Life; Linden Lab, San Francisco, California). Discussion forums were also included as they incorporate content that is publicly available and created by end users, and were judged to fall within the social media spectrum. We defined *social media in dietetic practice* as any social media platforms written by RDs for nutrition- and food-related purposes. Involvement of RDs with social media (eg, writing blog postings on positive messages to promote dietary behavior change or moderating a Facebook-based peer support group in a weight loss intervention) had to be specified in the study methods, or this information had to be obtained upon correspondence the authors. Studies were eligible regardless of their experimental design, users, and the degree of involvement of RDs with social media. We excluded studies in which the social media platform was not clearly described, studies on other eHealth technologies (eg, mobile apps), editorials, and publications not written in English or French.

### Stage 2: Identifying Studies and the Gray Literature

With the collaboration of a medical information specialist, we developed a search strategy to identify all relevant sources of information on social media in dietetic practice. Using specific keywords related to social media, Web 2.0, and nutrition, we conducted a systematic search, using November 15, 2016, as a cutoff date, in the following scientific databases: MEDLINE, EMBASE, PsycINFO, the Cochrane Library, Web of Science, ABI/INFORM Global, and ProQuest Dissertations & Theses. All databases were searched with a publication date range limit of 2000 or later, corresponding to the advent of social software and Web 2.0 applications [26]. The Medline search strategy is presented in Multimedia Appendix 1. This search strategy was thereafter modified to account for specificities of the other scientific databases.

We conducted additional searches by scanning the reference lists of included studies, exploring the literature with the search engine “Google scholar,” and searching for gray literature using the most widely used Internet search engines “Google,” “Bing,” and “Yahoo.” For each of these search engines, we used a more specific search string query. As performed by Archambault et al [27], we analyzed the first 100 results of each search engine, which displayed results by relative importance of website pages using a link analysis algorithm [28].

### Stage 3: Selecting Studies and the Gray Literature

Two review authors (AL and A-AD) independently assessed the eligibility of publications identified by the search strategies using titles and abstracts. Then, the same 2 reviewers retrieved full-text copies of publications that were judged potentially relevant to the review to validate inclusion. Disagreements were resolved through discussion and with a third review author (SD) when consensus was not reached. Authors were contacted to obtain further details when papers contained insufficient information to make a decision about eligibility.

### Stage 4: Charting the Data

A data-charting template was developed to extract the following common features from all studies: authors’ names, year of publication, title, journal, status of publication (eg, published, in press, or gray literature), country, experimental design, aim of the study, number of users, sociodemographic characteristics of users, type of social media studied, uses of social media, nutrition- and food-related outcomes studied, description of the effects of social media on outcomes studied, and description of barriers and facilitators that could affect the use of social media. The template was a priori tested with 10 included studies to validate extensiveness and clarity among the reviewers. The review authors independently extracted the data from all included studies and resolved any discrepancies in judgment by discussion and consensus, or with the third review author (SD) when necessary.

### Stage 5: Collating, Summarizing, and Reporting Results

As suggested by Levac et al [25], our analysis involved textual descriptions and data tables to map and summarize extracted data. To structure the presentation of results, we classified studies according to their research objectives: intervention studies (eg, studies investigating the effects of social media), descriptive studies (eg, studies describing who uses social media and for what purposes), content analysis studies (eg, studies in which information of social media content is analyzed), or expert opinion papers (eg, studies discussing ethical and professional use of social media by RDs).

A descriptive numerical summary of the study characteristics extracted was then conducted. Our classification for purposes of social media use was inspired by Coulter and Ellins’s classification scheme for patient-oriented interventions [29,30] with the addition of relevant dietetic, professional [31], and interactive technology [32] outcomes. Studies globally assessed multiple food- and nutrition-related outcomes and/or evaluated those outcomes at different times (eg, 16 weeks, 6 months, 12 months). Consequently, we retrieved all effects of social media on food- and nutrition-related outcomes as they were reported by authors in studies where intervention groups exposed to single or multiple social media platforms were compared with a control group with no social media access.

Finally, we performed a qualitative thematic analysis to identify potential barriers and facilitators related to the use of social media by users. The qualitative analysis was performed with the NVivo software, version 10 (QSR International, Cambridge, MA, 2012), and consisted of interpreting textual data subjectively by classifying and coding the information into categories that best reflected outputs we had identified [33]. The description of barriers and facilitators was guided by the validated taxonomy developed by Gagnon et al [31]. The review authors independently read each study and identified sentences or paragraphs in the text relevant to these categories and aggregated them into main themes to facilitate the synthesis. The review authors resolved any coding discrepancies through discussion and consensus.

### Stage 6: Consulting Knowledge Users

At each critical stage of the review process, we either held a teleconference meeting or exchanged emails with two RD representatives working, respectively, in public health nutrition and in a national dietetic professional association to explain our methodology and progression of our work and to gather their feedback and generate relevant results for dietetic practice.

## Results

### Description of Included Studies

**Figure 1:**
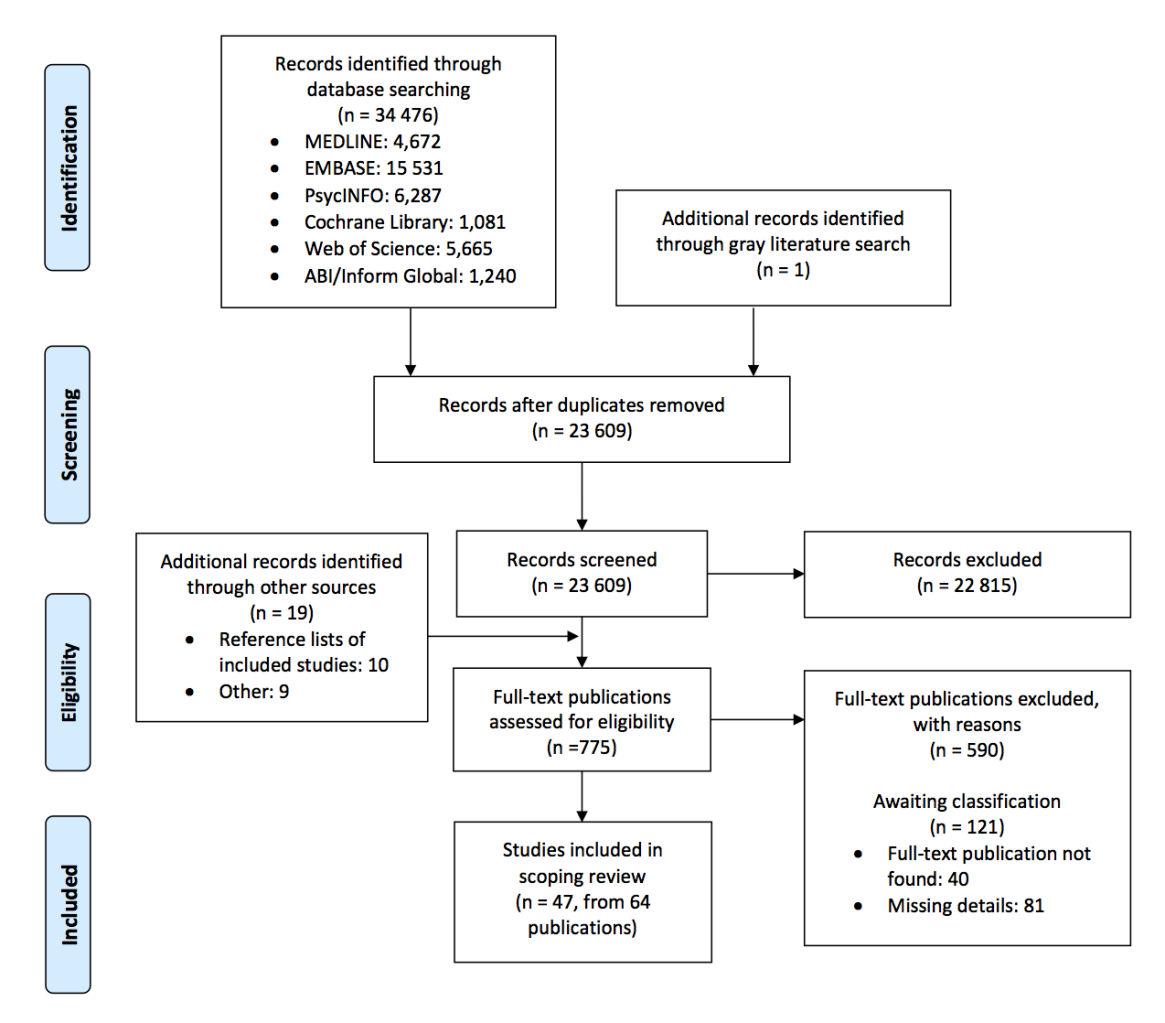
Preferred Reporting Items for Systematic Reviews and Meta-Analyses flow diagram for the scoping review process.

After excluding duplicates, we identified 23,609 potentially relevant publications from electronic databases and gray literature searches. From these, we excluded 22,815 publications after examining the titles and abstracts, and we retrieved 756 full texts of potentially relevant publications for detailed evaluation. During this screening process, we retrieved 19 additional publications from reference lists of included studies and other sources (the *Journal of the Academy of Nutrition and Dietetics* and authors’ contacts), for a total of 775 full-text publications assessed for eligibility. From these, 590 publications were excluded as at least one of our inclusion criteria was not met, and 121 publications were classified as awaiting classification due to our inability to locate full text or due to missing details despite attempts to contact study authors. A total of 64 publications (describing 47 unique studies) fulfilled our eligibility criteria and were included in this scoping review [16,18,19,34-94] (Figure 1).

**Table 1:** Distribution of included studies by country (N=47).

Country	Number of studies, n (%)	Studies
United States	26 (55)	[19,34,36,41,47,48,52-54,58,72-84,87,88,93]
Australia	6 (13)	[37,40,44,55,56,92]
Canada	5 (11)	[18,38,49,50,61]
Austria	2 (4)	[59,60]
Belgium	1 (2)	[43]
Germany	1 (2)	[63]
Ireland	1 (2)	[64]
Italy	1 (2)	[39]
Korea	1 (2)	[42]
United Kingdom	1 (2)	[66]
United States and Norway	1 (2)	[35]
New Zealand	1 (2)	[86]

**Figure 2:**
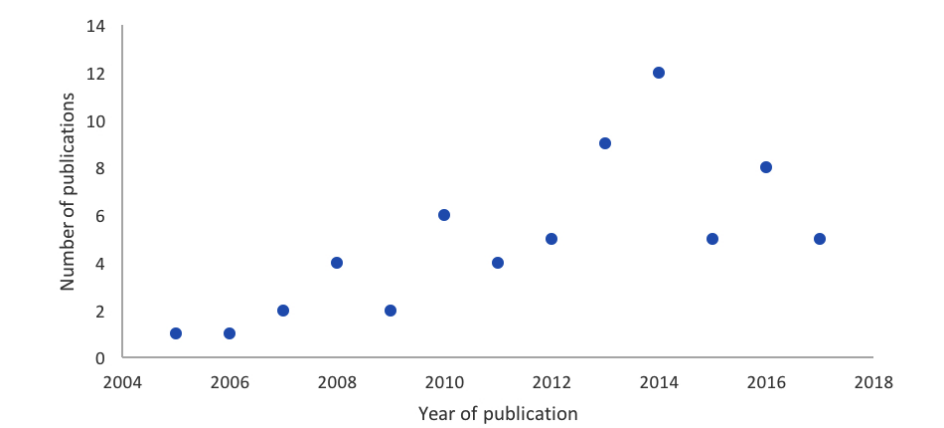
Distribution of included publications by years of publication (n=64).

The characteristics of included studies are summarized in Multimedia Appendix 2. Studies were categorized as intervention studies (n=34; eg, quasi-experimental or randomized control trials where intervention content was delivered through one or more social media platforms), descriptive studies (n=4; eg, qualitative studies reporting the design of social media or describing perceptions and preferences of social media users), content analysis studies (n=2; eg, studies analyzing the specific content of social media), or expert opinion papers (n=7; eg, Position Papers for Ethics in Practice published in the *Journal of the Academy of Nutrition and Dietetics*). Most publications reported primary research results (42/64; 66%). Of the publications, 10 were study protocols (10/64; 16%), 3 were conference abstracts (5%), and 2 were dissertations/theses (4%). Most studies were conducted in the United States, Australia, and Canada (Table 1).

Most studies were published from 2013 onward, with the highest number of publications occurring in 2014, 2013, and 2016 (Figure 2).

### Users of Social Media in Dietetic Practice

The majority of intervention studies targeted adult populations (26/34, 76%) [18,41-44,48,50,52,54-56,58-60,66,72,75,77,79, 82-84,87,88,92,93]. Among them, specific populations of users were adult women [18,50,66,84,88], young adults aged 18-35 years [55,56,72], pregnant adult women [79,92], and collegiate athletes [58]. RDs were the main users of social media in 2 intervention studies [47,78]. Remaining intervention studies targeted health care professionals (ie, those who expressed interest in enrolling in an online continuing nutrition education course [80] or professionals working in the fields of speech pathology, nursing, medical oncology, and pharmacy [40]), adolescents (2/34, 6%) [63,64], nuclear families with children aged 10-17 years (1/34, 3%) [37], and preschool-aged children and their parents (1/34, 3%) [76]. In descriptive studies, users of social media in dietetic practice were all adult populations [35,38,39,86], with some studies specifically targeting adult women [38] and RDs and patients [39]. RDs were the main users of social media in all expert opinion papers [19,34,36,53,73,74,81].

Intervention studies covered a limited range of health conditions, with most users of social media being overweight and obese (15/34, 44%) [41,44,52,54-56,59,60,72,75,82-84,88,92] or obese (3/34, 9%) [63,87,93]. In total, 8 intervention studies targeted healthy users (8/34, 24%) [18,43,50,58,66,76,79,80]. Other health conditions included patients with type 1 diabetes [64] (1/34, 3%), patients with type 2 diabetes [77] (1/34, 3%), patients with polycystic ovary syndrome (1/34, 3%) [84], and patients with metabolic syndrome (1/34, 3%) [42]. The principal health conditions of social media users were not described in 5 intervention studies [37,40,47,48,78]. Among descriptive studies, users of social media in dietetic practice were patients with type 1 or type 2 diabetes [35], healthy [38], or overweight and obese [86]. One descriptive study did not describe the health condition of social media users [39].

### Uses of Social Media in Dietetic Practice

**Figure 3:**
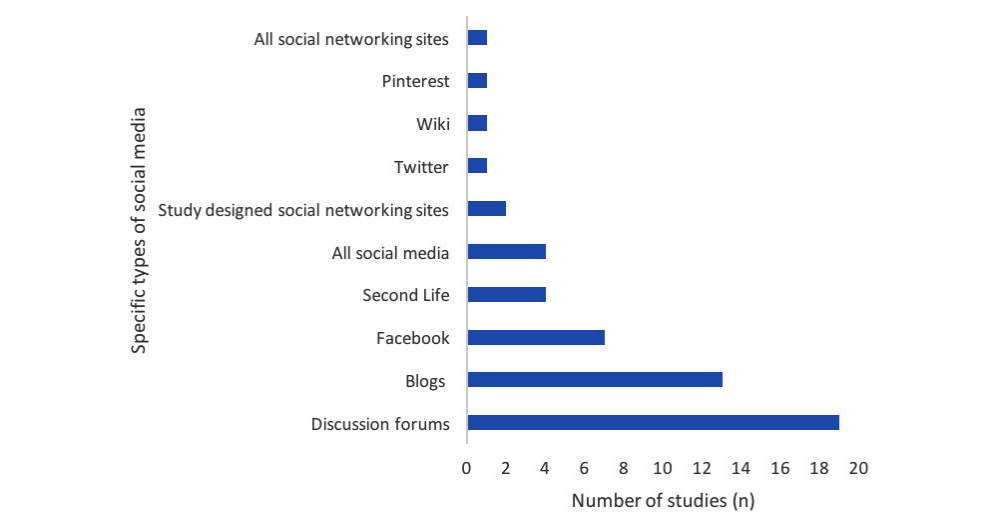
Frequency of social media tools evaluated in included studies (n=47).

Figure 3 illustrates the frequency of social media platforms evaluated in included studies. In this figure, *All social networking sites* refers to social networking sites that could be used for dietetic professional networking, such as LinkedIn and Facebook, as described by Graham 2009 [53], and *All social media* refers to all social media platforms (ie, blogs/microblogs, discussion forms, social networking sites, collaborative projects, content communities, and virtual worlds). In Figure 3, percentages do not add up to 100 due to the possibility of multiple social media platforms per study: the SMART study [51,72] included a social networking site (Facebook) and a blog; the study described in Baghaei 2011 [37] included a study designed social networking site entitled SOcial Families, a blog, and a discussion forum; and the study described in Hales (2014) [54] and Turner-McGrievy (2014) [84] included a social networking site (Facebook) and a microblog (Twitter). 

Discussion forums were the most frequent social media platforms evaluated, followed by blogs and the social networking site Facebook. In the majority of intervention studies (27/34, 79%) [37,41-43,44,48,52,54,55,56,58-60,63,66,72, 75-77,79,80,83, 84,87,88,92,93], the social media platform was part of a multicomponent intervention including other modes of delivery such as emails, websites, phone calls, text messaging, or face-to-face meetings. We identified 7 single-component intervention studies. Those studies, evaluated the effects of a blog for healthy eating behavior change among adult women [18,50], a closed Facebook group for diabetes management among adolescents with type 1 diabetes [64], a discussion forum for the delivery of online journal clubs among RDs [78], the virtual world Second Life for weight management among overweight or obese adults [82] and as a training tool for RDs to perform the subjective global assessment [47], and a wiki to disseminate evidence-based practice guidelines for the nutritional management of patients with head and neck cancer [40].

The main overarching research themes of included studies are displayed in Table 2. Among intervention studies, blogs or microblogs, discussion forums, social networking sites (eg, Facebook), and virtual social worlds (eg, Second Life) were specific social media platforms used to promote healthy eating, physical activity, or lifestyle behavior change. Descriptive studies focused mostly on users’ perceptions regarding the use of blogs to improve their dietary behaviors [38] and support self-monitoring for diabetes management [35] and users’ design preferences for a weight management program that included a blog [86]. Content analysis studies provided overviews of food blogs. Ethical and professional use of social media platforms by RDs was the main use of social media discussed in all expert opinion papers.

The specific contexts of use of social media in intervention studies are displayed in Table 3. Among intervention studies, social media platforms were most commonly evaluated in the contexts of weight management and diet, such as healthy eating promotion among French-Canadian women [18,50] and collegiate athletes living in the United States [58], or the promotion of the Mediterranean diet among adult Scottish women [66].

### Effects of Interventions Using Social Media in Dietetic Practice on Food- and Nutrition-Related Outcomes

Multimedia Appendix 3 presents the outcomes assessed in intervention studies using social media comparing single or multiple intervention groups with a control group with no social media access. Those studies evaluated blogs [18,55,58], discussion forums [44,48,56,66,75,78-80], a combination of Facebook and a blog [72] or a microblog (Twitter) [54], and a virtual social world (Second Life) [82]. Globally, study authors reported intervention effects on outcomes related to users’ health behaviors and status (eg, dietary intakes, body weight, and clinical indicators), compliance, participation and retention rates, and professional knowledge and self-efficacy.

Those effects were mostly neutral, but some authors reported positive or mixed effects (Multimedia Appendix 3). One study [46] reported a negative effect, with levels of eating restraint significantly higher in the intervention groups (exposed to a multicomponent behavior change intervention that included a discussion forum) compared with the control group after a 12-week Web-based weight loss intervention.

**Table 2:** Distribution of included studies according to main uses of social media (N=47).

Main use and types of social media^a,b^				Number of studies		Studies	
**Intervention studies**								
	**Promoting behavior change**						
		Blogs or microblogs		8		[18,37,50,54,55,58,72,76]	
		Discussion forums		15		[37,42-44,48,52,56,59,60,63,66,75,79,87,88]	
		Social networking sites		8		[37,41,54,64,72,84,92,93]	
		Virtual worlds		3		[77,82,83]	
	**Professional dietetic education**						
		Collaborative projects		1		[40]	
		Discussion forums		2		[78,80]	
		Virtual worlds		1		[47]	
**Descriptive studies**								
	**Promoting behavior change**							
		Blogs or microblogs		3		[35,38,86]	
		Social networking sites		1		[39]	
**Content analysis studies**								
	**Overview of social media content**							
		Blogs or microblogs		2		[49,61]	
**Expert opinion papers**								
	**Professional dietetic practice**							
		All social media		4		[19,34,36,74]	
		All social networking sites		1		[53]	
		Content communities		1		[73]	
		Discussion forums		1		[81]	

^a^All social networking sites” refers to social networking sites that could be used for dietetic professional networking, such as LinkedIn and Facebook, as described by Graham 2009 [53]; “all social media” refers to all social media platforms (ie, blogs/microblogs, discussion forms, social networking sites, collaborative projects, content communities, and virtual worlds).

^b^There was a possibility of multiple social media platforms per study: the Social Mobile Approaches to Reduce weighT (SMART) study. SMART study [51,72] included a social networking site (Facebook) and a blog; the study described in Baghaei 2011 [37] included a study designed social networking site entitled SOcial Families, a blog, and a discussion forum; and the study described in Hales (2014) [54] and Turner-McGrievy (2014) [84] included a social networking site (Facebook) and a microblog (Twitter).

Regarding positive effects, groups exposed to intervention content delivered through a social media platform (a blog, the virtual world Second Life, Facebook, or a discussion forum) had higher compliance [18,54], participation [18], and retention rates [18]; had significant improvements in vegetables [18] and fruit intakes [18,82]; were more satisfied with the intervention materials [66]; and had a higher increase in professional knowledge, skills, and self-efficacy in using an ecological approach to prevent childhood obesity among community-based nutrition and health professionals [80] compared with control groups with no social media exposure. In studies where interventions included a social media platform (a blog or a discussion forum) for peer support, positive effects were described for general nutrition knowledge [58], vegetables [55], fruits [82] and ultraprocessed food [55] intakes, body weight [44,55], cholesterol and blood pressure levels [44], and retention rates [44] among intervention groups compared with controls. Only one single-component study isolated and reported the specific effects of one social media. In this study [78], no statistically significant difference between a face-to-face group and a group of RDs participating in a Web-based journal club delivered through a discussion forum was found on users’ perceptions regarding the journal club environment (in terms of ability to meet the journal club objectives), the process of learning (in terms of critical appraisal skills), and the potential to apply knowledge to practice, and on users’ mean scores for the knowledge questions related to the study discussed in the journal club.

However, it was reported that RDs participating in the online journal club using a discussion forum had more positive perceptions of the journal club environment in terms of logistics for timing and opportunities for critical appraisal and of the process of learning in terms of discussion participation compared with the face-to-face control group.

**Table 3:** Distribution of included studies according to specific contexts of use of social media in intervention studies (N=34).

Contexts of use of social media		Number of studies	Studies
**Weight management**			
	Weight loss	12	[41,44,52,54,56,59,60,72,84,87,88,93]
	Weight loss and weight management	2	[82,83]
	Prevention of weight gain	3	[55,63,75]
	Prevention of pediatric obesity	1	[76]
	Prevention of excessive gestational weight gain	2	[79,92]
Healthy diet		4	[18,50,58,66]
Continuing professional education		3	[47,78,80]
Diabetes management		2	[64,77]
Healthy lifestyle		2	[37,48]
Cancer management		1	[40]
Cardiovascular disease prevention		1	[43]
Metabolic syndrome prevention		1	[42]

In studies using multiple intervention components, social media such as blog features [37,55,58], Facebook Fan Page [41], and discussion forums [48,56] were seldom accessed or used by study participants during the course of interventions to assist behavior change. On the other hand, an interesting finding reported by Patrick et al [72] was that “...Facebook emerged as the primary modality through which dynamic content was delivered at the group level” in the Social Mobile Approaches to Reduce weighT study.

In total, 6 multiple component intervention studies reported process measures relating to social media usage. Among those, Baghaei et al [37] found that increased engagement of families in lifestyle behavior change through social networking was associated with a decrease in users' perception that health was determined by external factors, such as chance. Gold et al [52] observed that the use of the discussion board feature was negatively correlated with weight change from baseline to 6 months among some intervention participants, but no association between the use of the discussion forum and weight change was observed during weight maintenance phase (6-12 months) of the study. In the Webber et al study [88], the number of publications submitted to the discussion forum was positively associated with weight loss. Hales et al [54] and Turner-McGrievy et al [84] both observed that engagement with Facebook (assessed by the number of views, likes, comments, and participant-initiated posts) was significantly associated with weight loss at 6 months. Finally, Karpinski 2012 [58] found a weak positive correlation between the number of blog postings (type of postings not described) and dietary behavior scores, but no association with self-efficacy scores among study participants.

### Barriers and Facilitators That Could Affect the Use of Social Media in Dietetic Practice by Users

#### Facilitators

A total of 5 studies [35,38,41,61,78] identified factors facilitating blog, discussion forum, or Facebook adoption by users (Table 4). Using Gagnon et al taxonomy [31], facilitators were mostly related to users' perceptions of the characteristics of the specific social media, such as *design and technical concerns* (eg, reminders of new posts via email), the *characteristics of the innovation* (eg, ease of use with quick access to desired information), and the *validity of the resources* (ie, appropriateness for the users and completeness of the information available) and, to a lesser extent, to *factors associated with social media users*. For example, the possibility to ask questions to the RD was a perceived facilitator for the use of healthy eating blogs written by RDs [38], and the presence of moderators’ post was cited as a facilitator to using Facebook in a weight management intervention [41]. Social support experienced with fellow social media users was an important facilitator for the use of healthy eating blogs by RDs [38], and for the participation of users in food-blogging communities, as several bloggers speaking of “the ‘comfort,’ ’encouragement,” and ‘supportive’ nature of the food-blogging community...” [61].

#### Barriers

A total of 6 studies [35,38,41,61,68,78] identified barriers to blog, discussion forum, or Facebook adoption by users. Globally, barriers were related to users' perceptions of the characteristics of the specific social media such as *the characteristics of the innovation* (eg, lack of usefulness of the social media for routine use or complicated access due to login identification) and *environmental issues* (eg, “the intimidation of online environment” in the context of online journal clubs [78], computer issues [38], and limited access to the Internet [41,78]). To a lesser extent, barriers were related to individual factors such as lack of time. For example, “being busy with life, going on vacation, and engaging in other family commitments” were barriers to participation in food-blogging communities [61]. Lack of time was also cited as a barrier to using Facebook, with participants mentioning they “had hectic lives and work schedules that interfered with intervention participation and behavior change” [41].

**Table 4:** Barriers and facilitators related to the use of social media in dietetic practice.

Factors (Gagnon et al taxonomy [31])^a^				Number of studies in which the factor was mentioned as a facilitator		Number of studies in which the factor was mentioned as a barrier	
**Factors related to the specific social media**							
	**Design and technical concerns**						
		Reminders^b^		3 [38,41,78]		1 [38]	
		Visual appearance^b^		1 [38]		1 [38]	
		Writing style^b^		1 [38]		1 [38]	
		Accessibility^b^		1 [38]			
	**Characteristics of the innovation**						
		Relative advantage (usefulness)				1 [35]	
		**Ease of use/complexity**					
			General ease of use/complexity of the social media platform^b^			1 [38]	
			Rapid/lengthy access to the social media platform^b^	1 [38]		2 [66,78]	
		Popularity of the social media site or of the author^b^		1 [38]			
	**Legal issues**						
		Conflict of interest, promotion of commercial products^b^				1 [38]	
	**Validity of the resources**						
		Scientific quality of the information resources				1 [38]	
		Content available (completeness)		1 [38]		1 [38]	
		Appropriate for the users (relevance)		1 [38]			
	**Environmental issues**						
		General online environment^b^				1 [78]	
		Computer issues^b^				1 [38]	
		Access to the Internet/limited access to the Internet^b^				2 [41,78]	
**Individual factors or health care professional characteristics (knowledge and attitude)**							
	Lack of time^b^						3 [38,41,61]
**Human environment**							
	**Factors associated with social media users**						
		Social media users/registered dietitian interaction		3 [35,38,41]			
		**Other factors associated with social media users**					
			Identification of other social media users^b^	1 [41]			
			Social media users should log in at the same time^b^	1 [78]			
			Requirement to respond to other social media users’ posts^b^	1 [78]			
			Social support from other users^b^	2 [38,61]			

^a^The following modifications were made to the Gagnon et al taxonomy [31] to fit the context of social media in dietetic practice: the term “Information and Communication Technologies (ICT)” was replaced with “social media,” the term “patients” was replaced with “social media users,” and the term “health professional” was replaced with “registered dietitian.”

^b^These new factors did not exist in the Gagnon et al taxonomy [31].

## Discussion

### Principal Findings

Using a scoping review methodology, we aimed to systematically map the literature available on social media in dietetic practice and to identify knowledge gaps. We found that this literature is relatively young but that it is growing fast. Most of the research results in this field have been published from 2013 onward. We retrieved 10 study protocols; therefore, new evidence can be expected in the near future. So far, research targeting social media written by RDs for diet and food-related purposes consisted mostly of experimental (eg, randomized controlled trials) and quasi-experimental studies in the context of weight management (ie, weight loss, prevention of weight gain, and prevention of unhealthy gestational weight gain) among overweight or obese adult users.

Although we can sense a growing interest among dietetic professional associations to promote an ethical and professional use of social media by RDs to improve knowledge translation in nutrition (7 expert opinion papers were published in the *Journal of the Academy of Nutrition and Dietetics*), we were intrigued to find only 4 intervention studies targeting RDs as social media users. Those studies were conducted in limited contexts of social media use (ie, continuing professional education and knowledge translation of evidence-based practice guidelines). There is also limited evidence of RDs' perspectives regarding the barriers and facilitators to the use of social media. From the perspective of lay users, the interaction with an RD through social media was mentioned as an essential facilitator to their behavior change process. However, we have yet to understand what constitutes quality exchanges between users and RDs through social media, how much bidirectional interaction is needed between users and RDs to provide clinically significant changes in dietary behaviors and outcomes, and what are RDs’ perspectives in those communications. Globally, research aiming at identifying adoption factors of social media in dietetic practice has only focused on healthy eating blogs, discussion forums, and Facebook. More research is needed on barriers and facilitators related to the use of other social media platforms such as collaborative projects (eg, wikis), virtual social worlds, and content communities (eg, Pinterest, YouTube), and how to make these tools useful for RDs to reach patients and health consumers.

So far, research on social media in dietetic practice has globally aimed to address, with only a few exceptions, one main question: *Are social media effective tools to promote dietary, physical activity, or lifestyle-related behavior change?* However, more work will be needed to provide a clear answer to this question. In general, neutral effects of the use of social media in dietetic practice on outcomes such as users’ health behaviors and status (eg, dietary intakes, body weight, and clinical indicators), compliance, participation and retention rates, and professional knowledge and self-efficacy have been reported in the literature. In concordance with a scoping review of social media use among patients and caregivers [95], these findings were mostly drawn from complex interventions where social media platforms were one component among various others, such as emails, interactive websites, and face-to-face consultations, for peer and counselor support in healthy behavior change. Few types of social media platforms have been evaluated or compared. Most intervention studies evaluated discussion forums, which are the oldest forms of social media and have the lowest scores in respect to social presence and media richness, as they are text-based and hence only allow for simple exchanges [7]. We found no study conducted uniquely with social media platforms such as Instagram, which has emerged as a popular tool to share food-related pictures [96] and convey social media norms regarding healthy eating [97,98], or Twitter, which has been recognized as a useful channel for the sharing and dissemination of health information [99,100]. Therefore, although best practices for the evaluation of the effectiveness of social media remains a debated question among behavioral research scientists [101], more research is needed to draw clear conclusions regarding the effectiveness of social media in dietetic practice and their mechanisms of action to support cost-effective and clinically significant behavior change.

This scoping review highlights a number of important knowledge gaps in the literature. As common difficulties in Web-based interventions include low actual reach, declined usage of online tools, and high attrition rates [102], there is a need for collaborative research and participatory action research to sustain a meaningful engagement of knowledge users. We have found only 4 studies addressing users’ salient beliefs and perceptions to design evidence-informed social media platforms for healthy behavior change. Many RDs working fields and dietetic-related outcomes have not yet been portrayed in the social media scientific literature. For example, the use of social media in the fields of child-feeding behaviors, food skills self-efficacy and acquisition, and the dissemination and implementation of social media-based nutrition interventions are yet to be investigated. As opposed to other fields in health care, such as medicine [103-105] and online health communities [106] for which content analyses of social media tools have been previously published, we only identified 2 content analysis studies of social media written by RDs and both focused on food blogs. Further comparative content analysis of social media written by RDs compared with layperson would help deepen our understanding of the quality and extent of nutrition information disseminated through social media. In addition, despite the unprecedented growth in the popularity of social media worldwide [107], recent studies have highlighted social inequalities in health, notably older and less educated individuals who represent an important percentage of the population who uses the Internet for health purposes [108,109]. Most of the evidence regarding the effectiveness and the use of social media in dietetic practice is based on adult populations living in developed countries such as the United States, Australia, and Canada, thus limiting the generalization of the results to other populations. Finally, women were the target population in most of the studies included in this review. It is now well recognized that women and men differ in their dietary intakes, eating behaviors, and meal preparation and cooking skills [110-113], and previous studies have identified gender differences on specific social media platforms usage (ie, women are more likely than men to use Pinterest, Facebook, and Instagram [114] and health forums [115]). Thus, there is a need for more research on gender-sensitive dietary interventions delivered through social media.

### Limitations

This scoping review was subject to some limitations that must be acknowledged. First, as performed in previous scoping reviews of social media use in health care settings [95,116], we categorized studies according to Kaplan and Haenlein’s social media definition [7], and we thought it was important to also include discussion forums as they represent the earliest form of user-generated content online. This methodological consideration orients the conclusions that can be drawn from this review. Second, despite an exhaustive search in relevant scientific databases and the reference lists of the identified studies as well as the gray literature, we cannot exclude the possibility that we missed some studies. Third, we included only studies written in English or French for time and budget constraints. Finally, given the fast-growing adoption of social media by health care professionals [9,10], we anticipate that the social media in dietetic practice literature will expand exponentially; this scoping review is limited to peer-reviewed studies or gray literature published before November 2016 (with the addition of one study [94]).

### Conclusions

Research on social media in dietetic practice is at its infancy, but it is growing fast. So far, this field of research has targeted limited social media platforms (ie, discussion forums, blogs, and Facebook), which were mostly evaluated in multiple-component interventions for weight management among overweight or obese adults. Trials isolating the effects and mechanisms of action of specific social media platforms are needed to draw clear conclusions regarding the effectiveness of those tools to support cost-effective and clinically significant behavior change. More work is also needed on barriers and facilitators underlying the use of social media written by RDs, and how to make these tools useful for RDs to reach patients and health consumers with diverse sociodemographic characteristics to improve dietary behaviors and help reduce social inequalities in health.

## Appendix

Multimedia Appendix 1 Medline search term strategy and number of results (November 2016).

Multimedia Appendix 2 Characteristics of included studies.

Multimedia Appendix 3 Types of outcomes assessed in intervention studies using social media comparing single or multiple intervention groups with a control group with no social media access (N=14 studies).

## Abbreviations

RD: registered dietitian
SMART: Social Mobile Approaches to Reduce weighT
