# Supplementary material for: Users, Uses, and Effects of Social Media in Dietetic Practice: Scoping Review of the Quantitative and Qualitative Evidence
Source: J Med Internet Res. 2018 Feb 20;20(2):e55. doi: 10.2196/jmir.9230 (PMC5840482; doi:10.2196/jmir.9230)
Supplement: Multimedia Appendix 1 [file jmir_v20i2e55_app1.pdf]

Multimedia Appendix 1. Medline search term strategy and number (n) of results

Search strategies developed by Annie Lapointe and Audrée-Anne Dumas in collaboration with the trial coordinator from the library of Laval University Daniela Zavala Mora.

Medline (November 15, 2016: n=4672 results)

#1 Social media
(internet [MH] OR social media [MH] OR crowdsourcing [MH] OR blogging [MH] OR "Mobile Applications"[MH] OR "Cell Phones"[MH] OR "web 2.0" [TIAB] OR "web2.0" [TIAB] OR "web 3.0" [TIAB] OR "web3.0" [TIAB] OR "social media" [TIAB] OR "social technology" [TIAB] OR "social media-based" [TIAB] OR "ehealth" [TIAB] OR "e-health" [TIAB] OR "health2.0" [TIAB] OR "health 2.0" [TIAB] OR "medicine2.0" [TIAB] OR "medicine 2.0" [TIAB] OR "social software" [TIAB] OR "social web" [TIAB] OR "web-based" [TIAB] OR "user generated content" [TIAB] OR "user-generated content" [TIAB] OR "internet" [TIAB] OR "wiki" [TIAB] OR "wikis" [TIAB] OR "collaborative and writing" [TIAB] OR "collaborative technology" [TIAB] OR "collaborative technologies" [TIAB] OR "collaborative software" [TIAB] OR "collaborative softwares" [TIAB] OR "quora" [TIAB] OR "medpedia" [TIAB] OR "social bookmarking" [TIAB] OR "delicious" [TIAB] OR blog* [TIAB] OR weblog* [TIAB] OR vlog* [TIAB] OR microblog* [TIAB] OR micro-blog* [TIAB] OR "twitter" [TIAB] OR "tweet" [TIAB] OR "tweets" [TIAB] OR "retweet" [TIAB] OR "hashtag" [TIAB] OR "hashtags" [TIAB] OR "tumblr" [TIAB] OR "sina weibo" [TIAB] OR "tencent weibo" [TIAB] OR "online community" [TIAB] OR "online communities" [TIAB] OR "online patient communities" [TIAB] OR "bookcrossing" [TIAB] OR "flickr" [TIAB] OR "youtube" [TIAB] OR "ustream" [TIAB] OR "picsearch" [TIAB] OR "vine" [TIAB] OR "YY"[TIAB] OR "slideshare" [TIAB] OR "social network" [TIAB] OR "social networks" [TIAB] OR "social networking" [TIAB] OR "facebook" [TIAB] OR "pinterest" [TIAB] OR "snapchat" [TIAB] OR "QQ" [TIAB] OR "QZone" [TIAB] OR "instagram" [TIAB] OR "Vkontakte" [TIAB] OR "google" [TIAB] OR "google plus" [TIAB] OR "reddit" [TIAB] OR "myspace" [TIAB] OR "livejournal" [TIAB] OR "orkut" [TIAB] OR "badoo" [TIAB] OR "yammer" [TIAB] OR "mig33" [TIAB] OR "migme" [TIAB] OR "mixi" [TIAB] OR "foursquare" [TIAB] OR "four square" [TIAB] OR "professional network" [TIAB] OR "professional networks" [TIAB] OR "professional networking" [TIAB] OR "linkedin" [TIAB] OR "viadeo" [TIAB] OR "researchgate" [TIAB] OR forum* [TIAB] OR "message board" [TIAB] OR "message boards" [TIAB] OR "Baidu Tieba" [TIAB] OR "healthmap" [TIAB] OR "patientslikeme" [TIAB] OR "patients like me" [TIAB] OR "curetogether" [TIAB] OR "cure together" [TIAB] OR "smart patients" [TIAB] OR "smartpatients" [TIAB] OR "smart patient" [TIAB] OR "smartpatient" [TIAB] OR "tudiabetes" [TIAB] OR "caringbridge" [TIAB] OR "dailystrength" [TIAB] OR "sharecare" [TIAB] OR "collaborative filtering site" [TIAB] OR "collaborative filtering sites" [TIAB] OR "digg" [TIAB] OR "whatsapp" [TIAB] OR "mobile app" [TIAB] OR "mobile apps" [TIAB] OR "mobile application" [TIAB] OR "mobile applications" [TIAB] OR "cell phone" [TIAB] OR "Cell Phones" [TIAB] OR "Smartphone" [TIAB] OR "Smartphones" [TIAB] OR "Text Messaging" [TIAB] OR "facebook messenger" [TIAB] OR "wechat" [TIAB] OR "skype" [TIAB] OR "viber" [TIAB] OR "bbm" [TIAB] OR "black berry messenger" [TIAB] OR "telegram" [TIAB] OR "virtual game world" [TIAB] OR "virtual game worlds" [TIAB] OR "virtual social world" [TIAB] OR "virtual social worlds" [TIAB] OR "second life" [TIAB] OR "clinispace" [TIAB] OR "mashup" [TIAB] OR "RSS" [TIAB] OR "rich site summary" [TIAB] OR "really simple syndicat" [TIAB] OR "RSSfield" [TIAB] OR "new feed" [TIAB])

AND

#2 Nutrition
(diet, food and nutrition [MH] OR nutrition assessment [MH] OR nutrition policy [MH] OR nutrition therapy [MH] OR nutritional sciences [MH] OR nutrition surveys [MH] OR nutritionists [MH] OR "food habit" [TIAB] OR "food habits" [TIAB] OR nutrition [TIAB] OR nutrition* [TIAB] OR nutritional [TIAB]

OR diet [TIAB] OR diets [TIAB] OR dietary [TIAB] OR "feeding behavior" [TIAB] OR "feeding behaviors" [TIAB] OR "feeding behaviour" [TIAB] OR "feeding behaviours" [TIAB] OR "feeding practices" [TIAB] OR nutritionist* [TIAB] OR dietician* [TIAB] OR dietitian* [TIAB] OR "dietetic professional associations" [TIAB] OR "dietetic professional association" [TIAB])
