# Supplementary material for: Users, Uses, and Effects of Social Media in Dietetic Practice: Scoping Review of the Quantitative and Qualitative Evidence
Source: J Med Internet Res. 2018 Feb 20;20(2):e55. doi: 10.2196/jmir.9230 (PMC5840482; doi:10.2196/jmir.9230)
Supplement: Multimedia Appendix 2 [file jmir_v20i2e55_app2.pdf]

Multimedia Appendix 2. Characteristics of included studies

Study	Country	Design	Aim of the study	Type of social media	Uses of social media	Context of use of social media	Type of users
**Intervention studies (n=34)**							
**Baghaei, 2011** [30]	Australia	RCT— four arms, parallel	To explore the effect of providing both family and social support, through an online social networking system, on engagement with the system and change in attitude toward healthy lifestyles.	• Study designed social networking site,<br>• Discussion forum,<br>• Blog	• Self-management education on health, exercise and diet,<br>• Peer support,<br>• Counselor support,<br>• Self-monitoring,<br>• Write about thoughts and feelings	• Healthy lifestyle	• Nuclear families (two parents and two children aged 10–17 years)
**Brown, 2013** [33]	Australia	Mixed—Expert consensus and Web usage statistics	To describe how a wiki platform has been utilized for the development and dissemination of a set of evidence-based practice guidelines for the nutritional management of patients with head and neck cancer.	• Wiki	• Development and dissemination of evidence-based practice guidelines for nutrition interventions in primary care	• Cancer management	• Health care professionals<br>• Registered dietitians
**Caplette, 2017** [34]	Canada	Pilot—RCT—two armed, parallel	To assess the feasibility of a dietary intervention using a healthy eating blog written by a registered dietitian through collecting blog browsing history data for each participant to determine compliance rates, participation rates, and attrition rates.	• Blog	• Peer support, counselor support,<br>• Self-management education on healthy eating	• Healthy diet	• Healthy adult women
**Cavallo, 2016** [35]	USA	Pilot—Single group pre-post design	To examine the feasibility of delivering a previously tested group-based weight-loss intervention adapted to low-income women of reproductive age using Web-based educational content and social media.	• Facebook	• Peer support,<br>• Counselor support,<br>• Peer role modeling in diet and physical activity behavior change,<br>• Promote user	• Weight management—Weight loss	• Overweight/ obese adults

					engagement in the intervention		
**Choi, 2014** [36]	Korea	Pilot—Single group pre-post design	To develop a Web-based nutritional management program for the prevention of metabolic syndrome and to evaluate how the program affects health-related behaviors and the health status of office workers with metabolic syndrome risk factors.	• Discussion forum	• Peer support, counselor support,<br>• Self-management education on health, nutrition and exercise	• Metabolic syndrome prevention	• Adults with metabolic syndrome
**Claes, 2007** [37]	Belgium	RCT— two arms, parallel	To determine the effects of a computer-tailored cardiovascular prevention program with the general practitioner as a key figure.	• Discussion forum	• Peer support,<br>• Counselor support	• Cardiovascular disease prevention	• Healthy adults
**Collin, 2010** [38–40, 51, 59]	Australia	RCT— three arms, parallel	To examine the efficacy of a 12-week commercial Web-based weight-loss program versus a version of this program with additional online features and personalized e-feedback and reminder calls (enhanced) versus a wait-list control (control) on initial weight loss and maintenance of weight lost eight up to 18 months.	• Discussion forum	• Peer support	• Weight management—Weight loss	• Overweight/ obese adults
**Connery, 2012** [41]	USA	Qualitative—Individual interviews	To develop a free and effective tool to train clinicians on performing the subjective global assessment.	• Second life	• Professional education on performing the subjective global assessment	• Continuing professional education	• Registered dietitians
**Dickinson, 2013** [42]	USA	RCT— two arms, parallel	To describe the development, implementation, and effectiveness of 2 interactive websites designed to support health behavior change around healthy eating, physical activity, smoking, and use of alcohol for primary care patients.	• Discussion forum	• Peer support,<br>• Counselor support	• Healthy lifestyle	• Adults
**Dumas, 2017** [44]	Canada	Mixed—RCT, two arms, parallel	To describe the use of the Intervention Mapping protocol to develop an evidence-informed	• Blog	• Self-management education on healthy eating,	• Healthy diet	• Healthy adult women

		and focus groups	blog written by a registered dietitian—used as a knowledge translation tool—to promote healthy eating among French-Canadian mothers of preschool and school-aged children.		• Peer support,<br>• Counselor support		
**Gold, 2007** [46]	USA	Pilot—RCT—two arms, parallel	1- To compare weight losses achieved through an online behavioral intervention versus a commercial self-help website;<br>2- To evaluate the use of web components and their relationship to weight loss between groups and within groups to identify which web components correlated with weight loss.	• Discussion forum	• Peer support,<br>• Counselor support	• Weight management—Weight loss	• Overweight/ obese adults
**Hales, 2014** [48, 80]	USA	RCT— five arms, parallel	1- To examine which post types prompt the most engagement (e.g., responses) from weight loss study participants during a weight loss intervention utilizing Facebook for information delivery and social support and which posts do participants report they prefer.<br>2 - To assess differences in engagement among participants between counselor-initiated posts and participant-initiated posts.<br>3- To examine if engagement in social networking support groups during the maintenance phase of a weight loss study enhances weight loss.	• Facebook, Twitter	• Peer support, counselor support,<br>• Self-management education on weight loss, nutrition and cooking practices,<br>• Self-monitoring for weight loss	• Weight management—Weight loss	• Overweight/ obese adults
**Hebden, 2013** [49, 63–65]	Australia	Mixed—RCT—two arms, parallel and individual interviews	1- To test the efficacy of the TXT2BFiT program by comparing changes in body weight and selected dietary, physical activity and sedentary behaviors among young adults aged 18 to 35 years with changes in a control group.	• Blog	• Peer support,<br>• Counselor support	• Weight management—Prevention of weight gain	• Overweight/ obese or at high risk for development of obesity young adults aged 18–35 years

			2- To evaluate program reach, costs, implementation and participant engagement to inform the potential future translation of the program into the broader community.				
**Hebden, 2014** [50]	Australia	Pilot, RCT—two arms, parallel	To measure the effect of a mHealth intervention program on body weight, body mass index (BMI) and the specific lifestyle behaviors addressed by the program [i.e. physical activity and sedentary behavior, intake of fruit and vegetables, energy-dense takeaway meals and sugar-sweetened beverages (SSB)] compared to a control group and, second, to measure participants' engagement with program materials and processes related to program delivery.	• Discussion forum	• Peer support, counselor support,<br>• Self-management education on lifestyle behavior change associated with weight gain during young adulthood	• Weight management—weight management	• Overweight/ obese young adults aged 18–35 years
**Karpinski, 2012** [52]	USA	RCT— two arms, parallel	To examine the impact of a 9-week interactive Internet-based nutrition education program on sports nutrition knowledge, dietary behaviors, and self-efficacy of collegiate athletes.	• Blog	• Peer support,<br>• Counselor support	• Healthy diet	• Healthy collegiate athletes
**Longin, 2012** [53]	Austria	Retrospective, Single group pre-post design	To present a large collection of user data, to compare KiloCoach with other evaluated commercial weight-loss programs and to analyze aspects of the programs that could explain different results.	• Discussion forum	• Peer support,<br>• Counselor support	• Weight management—Weight loss	• Overweight/ obese adults
**Luger, 2016** [54]	Austria	Single group pre-post design	1- To characterize the dietary patterns of participants in a Web-based weight-reduction program, 2- To examine the weight-loss success associated with the distinct dietary patterns, 3—To determine the changes in these	• Discussion forum	• Peer support,<br>• Counselor support	• Weight management—Weight loss	• Overweight/ obese adults

			dietary patterns over time.				
**Markert, 2014** [57]	Germany	Pilot—RCT, three arms, parallel	1- To present the study concept for the TeAM program; 2—To present baseline data of the feasibility study.	• Discussion forum	• Peer support	• Weight management—maintaining weight loss	• Obese adolescents aged 14–18 years
**McDarby, 2015** [58]	Ireland	RCT— two arms, parallel	To assess the use of a social network site as a resource light intervention medium to increase diabetes knowledge, self-efficacy and glycemic management of adolescents with type 1 diabetes.	• Facebook	• Peer support,<br>• Counselor support,<br>• Self-management education on diabetes management	• Diabetes management	• Adolescents with type 1 diabetes
**Papadaki, 2005** [60–62]	United Kingdom	Mixed—Quasi-experimental, two arms, pre-post design and focus groups	To explore the effects on dietary behavior of a Web-based, stepwise, tailored-feedback nutrition intervention that promotes four key components of the traditional Mediterranean diet.	• Discussion forum	• Peer support	• Healthy diet	• Healthy adult women
**Patrick, 2014** [45, 66]	USA	RCT— two arms, parallel	To assess the efficacy of a 2 year, theory-based, weight loss intervention that was remotely and adaptively delivered via integrated user experiences with Facebook, mobile apps, text messaging, emails, a website, and technology-mediated communication with a health coach (the SMART intervention).	• Facebook,<br>• Blog	Facebook:<br>• Peer support,<br>• Counselor support,<br>• Self-management education for weight-related behavior change;<br>Blog:<br>• Peer support,<br>• Counselor support,<br>• Self-management education on weight management	• Weight management—Weight loss	• Overweight/ obese young adults aged 18–35 years
**Petersen, 2008** [69]	USA	Single group pre-post design	To evaluate an employee Internet-based weight management program offered to a large, dispersed employee population.	• Discussion forum	• Peer support,<br>• Counselor support,<br>• Self-management education on	• Weight management—weight management	• Overweight/ obese adults

					healthy eating and weight management		
**Po'e, 2013** [70]	USA	RCT— two arms, parallel	To evaluate the efficacy of a family-centered, behavioral intervention to prevent pediatric obesity among children ages 3–5.	• Blog	• Self-management education on nutrition, physical activity, and parenting skills,<br>• Peer support,<br>• Counselor support,<br>• Self-monitoring, support engagement of users in the intervention	• Weight management—Prevent pediatric obesity	• Healthy parent-child dyads
**Ruggiero, 2014** [71]	USA	Pilot—Single group pre-post design	1- To implement and evaluate the preliminary impact on self-care of a *Second Life* virtual world intervention ("Diabetes Island") designed to provide diabetes self-management education to facilitate optimal diabetes self-management in low-income African Americans with diabetes attending primary care clinics; 2- To purposefully recruit underserved individuals who might not have the opportunity to participate due to lack of computer/Internet skills and/or access	• Second Life	• Self-management education on diabetes management,<br>• Peer support,<br>• Counselor support,<br>• Promote user engagement in the intervention	• Diabetes management	• Adults with type 2 diabetes
**Schumacher, 2013** [72]	USA	Mixed—RCT, two arms parallel and qualitative data obtained from open-ended questions	To analyze the perceptions of the learning environment, the perceived learning, and the actual learning of dietetic professionals participating in online journal clubs, as compared to dietetic professionals participating in face-to-face clubs.	• Discussion forum	• Professional education,<br>• Professional networking	• Continuing professional education	• Registered dietitians

**Smith, 2016** [73]	USA	RCT— two arms, parallel	To determine if previously sedentary women utilizing a Web-based behavioral intervention designed to increase sustained physical activity would prevent excessive gestational weight gain.	• Discussion forum	• Peer support	• Weight management—Prevent excessive gestational weight gain	• Healthy pregnant adult women
**Stark, 2011** [74]	USA	Quasi-experimental, two arms	To evaluate the effectiveness of a facilitated online course to increase the knowledge, skills, and self-efficacy of community-based nutrition and health professionals to address childhood obesity using an ecological approach.	• Discussion forum	• Professional education on childhood obesity prevention	• Continuing professional education	• Health care professionals<br>• Registered dietitians
**Sullivan, 2011** [76, 78]	USA	Mixed—Pilot—RCT—two arms and individual interviews	To evaluate the efficacy of Second Life for weight loss and weight maintenance.	• Second Life	• Peer support,<br>• Counselor support,<br>• Self-management education on diet and physical activity behavior change	• Weight management—weight loss and weight maintenance	• Overweight/ obese adults
**Sullivan, 2016** [77]	USA	Mixed—Pilot—RCT—two arms and individual interviews	1- To compare between group differences in weight change during weight maintenance, 2—To assess potential mediators of weight change and conduct a qualitative and quantitative process analysis to monitor quality control, determine if the interventions were delivered as intended, assess challenges and barriers to intervention compliance, participant satisfaction, and potential competing factors that may have contaminated or diminished the effectiveness of the intervention.	• Second Life	• Peer support,<br>• Counselor support,<br>• Self-management education on diet and physical activity behavior change	• Weight management—weight loss and weight management	• Overweight/ obese adults
**Turner-McGrievy, 2014** [79]	USA	Pilot, RCT—two arms, parallel	To examine the effectiveness of two dietary approaches for weight loss among women with PCOS	• Facebook	• Peer support,<br>• Counselor	• Weight management—Weight loss	• Overweight/ obese adult women with

			who were trying to conceive: a low-fat, low—glycemic index vegan diet with no caloric restriction and a standard, low-cal diet.		support,<br>• Self-management education on weight loss		polycystic ovary syndrome
**Webber, 2008** [83–86]	USA	RCT— two arms, parallel	To investigate the efficacy of the addition of weekly motivational enhanced chats to a standard behavioral Web-based weight loss program.	• Discussion forum	• Not described	• Weight management—Weight loss	• Overweight/obese adult women
**Webber, 2013** [82]	USA	Pilot—RCT—two arms, parallel	1- To compare the efficacy of a 12-week Web-based behavioral weight loss program, prescribed with a diet of self-selected conventional foods (Internet-alone group), to the same 12-week program supplemented with a commercially available portion-controlled diet for producing weight loss (Internet + PCD);<br>2- To compare the impact of the two programs on chronic disease indicators including blood pressure, blood lipids, and blood glucose levels.	• Discussion forum	• Not described	• Weight management—Weight loss	• Obese adults
**Willcox, 2015** [87]	Australia	Pilot—RCT—two arms, parallel	To test the feasibility of an mHealth intervention to promote healthy nutrition, physical activity and weight gain in pregnant women who are overweight or obese prior to pregnancy.	• Facebook	• Self-management education on diet and physical activity behavior change,<br>• Peer support,<br>• Counselor support,<br>• Self-monitoring	• Weight management—Prevent excessive gestational weight gain	• Overweight/obese pregnant adult women
**Willis, 2016** [88]	USA	RCT— two arms, parallel	To compare weight loss between an established cost-effective weight management delivery system (group conference call) and online social network (Facebook) delivered weight management intervention.	• Facebook	• Peer support,<br>• Counselor support,<br>• Self-management education on diet and physical	• Weight management—Weight loss	• Obese adults

					activity behavior change		
**Descriptive studies (n=4)**							
**Arsand, 2008** [28]	Norway and USA	Qualitative—Individual interviews	To perform two cycles of laboratory-based usability testing of three food registration prototypes for people with diabetes.	• Blog	• Self-monitoring of carbohydrate intakes,<br>• Online nutritional counseling with a registered dietitian	• Diabetes management	• Adults with type 1 or type 2 diabetes
**Bissonnette-Maheux, 2015** [31, 89]	Canada	Qualitative—Individual interviews and focus groups	To identify women's salient beliefs and perceptions regarding the use and the characteristics of healthy eating blogs written by registered dietitians promoting the improvement of their dietary habits.	• Blog	• Self-management education on healthy eating	• Healthy diet	• Healthy adult women
**Bonacina, 2009** [32]	Italy	Descriptive	To report the design and the implementation of a dynamic web application for controlling eating and physical activity behaviors based on the cooperation between patients and registered dietitians.	• Study designed social networking site	• Peer support,<br>• Counselor support	• Healthy lifestyle	• Population,<br>• Registered dietitians
**Waterlander, 2014** [81]	New Zealand	Mixed—Expert consensus, individual interviews, focus groups and quantitative survey	To develop an evidence-based mHealth weight management program appropriate for ethnically and socioeconomically diverse target groups (Horizon), using a structured content development process (involving the target audience in the development stages) and formative research.	• Blog	• Peer support,<br>• Counselor support	• Weight management—Weight loss	• Overweight/ obese adults
**Content analysis studies (n=2)**							
**Dumas, 2017** [43]	Canada	Quantitative	To compare the nutritional content of vegetarian recipes published in food blogs written by registered dietitians and by laypeople.	• Blog	• Share recipes and individual's cooking practices	• Healthy diet	• Population,<br>• Registered dietitians
**Lynch, 2010** [55–56]	Canada	Qualitative	To describe the virtual socialization, nutritional	• Blog	• Self-monitoring of meals and snacks	• Healthy diet	• Young adult female

			behaviors, and attitudes being promoted in one community of healthy food bloggers.		consumed		bloggers
**Other types of studies (n=7)**							
**Aase, 2010** [27]	USA	Expert Opinion	Not described	• All	• Use of social media for the practice of dietetics	• Professional practice	• Registered dietitians
**Ayres, 2013** [29]	USA	Expert Opinion	To explore the benefits and risks of social media for the practice of dietetics and outlines professional and personal responsibilities for dietetics practitioners.	• All	• Use of social media for the practice of dietetics.	• Professional practice	• Registered dietitians
**Graham, 2009** [47]	USA	Expert Opinion	Not described	• All	• Professional networking	• Professional practice	• Registered dietitians
**Helm, 2016** [13]	USA	Expert Opinion	To provide the necessary guidance to help nutrition and dietetics practitioners engage confidently and responsibly in social media to: 1—position themselves as trusted sources of science-based information on multiple social media platforms; 2- increase their digital presence to positively impact the balance of accurate online information about food and nutrition; 3- broaden practice applications and seize new business opportunities enabled by social media; 4) and avoid mistakes and misjudgments involving ethics, professionalism, transparency, and disclosure.	• All	• Use of social media for the practice of dietetics.	• Professional practice	• Registered dietitians
**Peregrin, 2012** [67]	USA	Expert Opinion	Not described	• Pinterest	• Professional promotion and branding	• Professional practice	• Population,<br>• Registered dietitians
**Peregrin, 2017** [68]	USA	Expert Opinion	To describe the ethical and legal aspects of copyright and plagiarism specifically related to online and digital platforms,	• All	• Sharing or posting social media content	• Professional practice	• Registered dietitians

			outlines what nutrition and dietetics practitioners need to know when sharing or posting content created by someone else, and provides insights from legal experts for navigating the fair use provision in the US Copyright Act.				
**Stark, 2014** [75]	USA	Expert Opinion	To describe the design/development, implementation, and lessons learned from two existing massive online open courses used by registered dietitians to provide nutrition-related educational opportunities for global audiences — public and professional; To describe opportunities and challenges of massive online open courses in the dietetic practice.	• Discussion forum	• Peer support,<br>• Counselor support	• Professional practice	• Population<br>• Health professionals<br>• Registered dietitians
