# Supplementary material for: Users, Uses, and Effects of Social Media in Dietetic Practice: Scoping Review of the Quantitative and Qualitative Evidence
Source: J Med Internet Res. 2018 Feb 20;20(2):e55. doi: 10.2196/jmir.9230 (PMC5840482; doi:10.2196/jmir.9230)
Supplement: Multimedia Appendix 3 [file jmir_v20i2e55_app3.pdf]

Multimedia Appendix 3. Types of outcomes assessed in intervention studies using social media comparing single or multiple intervention groups with a control group with no social media access (N=14 studies).

Outcomes[a]	Number of studies in which the effect is reported as positive	Number of studies in which the effect is reported as neutral	Number of studies in which the effect is reported as negative	Number of studies in which the effect is reported as mixed
**Users' knowledge**				
**Self-care knowledge**				
General nutrition knowledge[b]	1 [58]			
Sports nutrition knowledge[b]		1 [58]		
**Users' experience of care**				
Quality of life		1 [48]		1 [44]
Self-efficacy		1 [58]		
**Users' health behaviors and status**				
**Health related lifestyles**				
**Dietary intakes[b]**				
Vegetables[b]	2 [18,55]	3 [56,66,82]		1 [44]
Fruits[b]	2 [18,82]	2 [55,56]		2 [44,66]
Grain products[b]		3 [18,44,66]		
Milk and alternatives[b]		2 [18,44]		1 [66]
Meat and alternatives[b]		2 [18,44]		1 [66]
Ultra-processed foods (eg, energy-dense takeaway meals, sugar-sweetened beverages)b	1 [55]	2 [56,82]		1 [44]
Total energy[b]				2 [44,79]
**Carbohydrates[b]**		2 [44,79]		

Dietary fibers[b]		1 [44]		
Sugar[b]		1 [44]		
Proteins[b]		2 [44,79]		
**Fat**[b]		2 [44,79]		
Saturated fat[b]		1 [44]		
Unsaturated fat[b]		1 [44]		
Monounsaturated fatty acids: saturated fatty acids ratio[b]		1 [66]		
Alcohol[b]		2 [44,66]		
Sodium[b]		1 [44]		
Diet quality (eg, Mediterranean score)[b]		2 [48,66]		
Dietary behaviors (eg, cognitive restraint, disinhibition)[b]			1 [46]	2 [44,58]
Physical activity[b]		2 [44,48]		4 [55,56,79,82]
Sedentary activity/sitting time[b]		2 [56,79]		
**Mental functioning**				
Depression symptoms[b]		1 [48]		
**Clinical indicators**				
**Body weight/body mass index**[b]	2 [44,55]	2 [18,56]		3 [72,75,82]
Gestational weight gain[b]		1 [79]		
Waist circumference[b]		1 [18]		1 [72]
Arm circumference[b]		1 [72]		
Total cholesterol[b]	1 [44]	1 [66]		
High-density lipoprotein (HDL) cholesterol[b]		2 [44,66]		1 [66]
Low-density lipoprotein (LDL) cholesterol[b]		2 [44,66]		
Total/HDL cholesterol ratio[b]				1 [66]

		Triglycerides[b]		2 [44,66]		
		Insulin[b]		1 [44]		
		Glucose/HbA1c[b]		1 [44]		
		Blood pressure[b]	1 [44]			1 [72]
		Heart rate[b]		2 [44,72]		
		Global health risk scores[b]		1 [48]		
**Others**[b]						
	Compliance rates[b]		2 [18,54]	1 [82]		
	Participation rates/usage statistics		1 [18]	1 [48]		
	Attrition/retention rates[b]		2 [18,44]	1 [54]		
	**User perspectives**					
		Intention to use				
		Satisfaction	1 [66]			
		Learning environment[b]				1 [78]
		Learning process[b]				1 [78]
		Application of professional knowledge[b]		1 [78]		
	Professional knowledge[b]		1 [80]	1 [78]		
	Professional self-efficacy[b]		1 [80]			
